# Improving Accessibility in the Emergency Department for Patients with Disabilities: A Qualitative Study

**DOI:** 10.5811/westjem.58406

**Published:** 2023-05-03

**Authors:** J. Harrison Carmichael, Kelly M. Kalagher, Martin A Reznek, Payal Modi

**Affiliations:** * University of Massachusetts, Chan Medical School, Worcester, Massachusetts; † University of Massachusetts, Chan Medical School, Department of Emergency Medicine, Worcester, Massachusetts

## Abstract

**Introduction:**

The emergency department (ED) is a critical service area for patients living with disabilities in the United States. Despite this, there is limited research on best practices from the patient experience regarding accommodation and accessibility for those with disabilities. In this study we investigate the ED experience from the perspective of patients living with physical and cognitive disability, as well as visual impairment and blindness, to better understand the barriers to accessibility in the ED for these populations.

**Methods:**

Twelve individuals with either physical or cognitive disabilities, visual impairments or blindness were interviewed regarding their ED experiences, particularly related to accessibility. Interviews were transcribed and coded for qualitative analysis with generation of significant themes relating to accessibility in the ED.

**Results:**

Major themes from coded analysis were as follows: 1) inadequate communication between staff and patients with visual impairments and physical disabilities; 2) the need for electronic delivery for after-visit summaries for individuals with cognitive and visual disabilities; 3) the importance of mindful listening and patience by healthcare staff; 4) the role of increased hospital support including greeters and volunteers; and 5) comprehensive training with both prehospital and hospital staff around assistive devices and services.

**Conclusion:**

This study serves as an important first step toward improving the ED environment to ensure accessibility and inclusivity for patients presenting with various types of disabilities. Implementing specific training, policies, and infrastructure changes may improve the experiences and healthcare of this population.

## INTRODUCTION

An estimated 61 million adults in the United States live with disability.^1^ Data from the 2006–2008 Medical Expenditure Panel Survey, a US health survey representative of community-dwelling civilians, demonstrated that people living with a disability accounted for roughly 40% of annual ED visits despite representing less than a quarter of the adult population.^2^ Factors such as complex medical profiles, poor access to medical care, and urgency of medical needs play important roles in contributing to the need for higher utilization among patients living with disabilities (PWD).^2^ Deaf/American Sign Language users and individuals living with autism are at a higher risk of using the emergency department (ED) than the general population,^3^,^4^ and adults of working age living with disabilities have higher rates of ED usage than individuals without disabilities.^1^

While some studies have explored the experiences of PWD in other healthcare settings such as primary care,^5^ Medicaid-managed care,^6^ general access to healthcare,^7^ hospital admissions and hospital care, and even as standardized patients,^8^–^13^ no studies to our knowledge have investigated experiences specific to the ED for these patients. Additionally, the majority of qualitative studies in alternate healthcare environments were performed in other Western countries with different healthcare systems compared to the US. The lack of research investigating the ED experiences of those living with disabilities represents a large gap in understanding between ED staff and these patient populations, which comprise a significant number of ED visits each year nationwide. In this study our goal was to understand the perspectives of patients living with various forms of disability as they access care in the ED, specifically identifying barriers and potential solutions to create an inclusive, accessible, patient-centered care environment.

## METHODS

### Study Criteria and Recruitment

From July 2021–July 2022, patients with disabilities were recruited through patient advocacy groups, advertisements on social media, contacts with local clinicians, or through word of mouth. Inclusion criteria included adults who had visited local EDs in the prior 18 months and were living with a disability including the following: significant visual impairment or vision loss; significant hearing impairment or deafness; mobility impairments; and autism or other intellectual and developmental disability. Participants were required to have access to the technology necessary for remote interviewing, such as a phone or laptop with video call capabilities. Exclusion criteria included those without the capacity to give informed consent or without the technology needed to conduct the interview. All potential participants were screened using a REDCap electronic data capture survey hosted at the University of Massachusetts to verify eligibility before scheduling an interview. This study was approved by the university’s institutional review board.

### Interviews and Analysis

Semi-structured interviews were conducted by study staff via video call using Zoom (Zoom Video Communications, San Jose, CA) or a telehealth platform (Caregility. Eatontown, NJ). Interviews lasted approximately 45–60 minutes each. Participants were mailed a $50 Visa gift card for their participation.

Each interview was audio recorded with consent from the participant and transcribed by the lead author. Each transcription was then deidentified and entered into qualitative data analysis software (Dedoose, Manhattan Beach, CA),^14^ for storage of the data, labeling of codes, and analysis of each transcript. In the initial coding phase, we reviewed transcripts using a grounded theory framework,^15^ which permitted the generation of codes informed by reviewing the available data to establish the initial codebook. After this initial phase, each interview transcript was then coded independently by two researchers. Throughout this process the codebook was continually updated with emerging codes derived from the data as similarities and differences between the transcript data were identified. Coding of the transcripts continued until analysis yielded no newly emerging codes, at which point it was determined that theoretical saturation had been reached. We then grouped the final codes into themes, which were refined through team discussions until the final five themes were determined.

**Population Health Research Capsule**

What do we already know about this issue?

*Little is known about how people with disabilities (PWD experience care in the ED. Research in other clinical contexts suggests a need for more inclusive environments*.

What was the research question?

*What are the experiences of PWD who have received care in the ED, and what barriers to inclusive care exist in this space?”*

What was the major finding of the study?

*Subjects described 1) inadequate communication between staff and patients; 2) the need for electronic delivery for after-visit summaries 3) the importance of mindful listening and patience by healthcare staff; 4) the need for increased hospital support including greeters and volunteers; and 5) comprehensive training with staff about assistive devices and services*.

How does this improve population health?

*We describe actionable changes that can be made to improve ED accessibility, with suggestions derived from the recommendations of PWD*.

## RESULTS

### Participants

Twelve participants were interviewed for this study. Participants had a mean age of 62 years, with 10 participants (83%) identifying as female. Four participants (33%) identified as Black and eight participants (66%) identified as White (Table 1). All participants were English-speaking. Six participants reported living with physical disability (50%), six reported visual impairment or blindness (50%), and two reported living with cognitive disability (16%). Several participants reported living with more than one disability and were encouraged to speak about the entirety of their experience.

### Themes

Five emergent themes were derived from the data. These included the following: 1) inadequate communication between staff and patients with visual impairments and physical disabilities; 2) the need for electronic delivery for after-visit summaries (AVS) for individuals with cognitive and visual disabilities; 3) the importance of mindful listening and patience by healthcare staff; 4) the role of increased hospital support services including greeters and volunteers; and 5) comprehensive training with both prehospital and hospital staff around assistive devices and services. Each of these themes is described in detail below, with specific quotes chosen that were deemed to be representative of the study results.

#### Inadequate communication between staff and patients with visual impairments and physical disabilities

Participants identified multiple communication gaps where staff lacked consideration when communicating with someone with a disability/impairment. Participants emphasized the importance of being properly addressed by name to help them navigate the healthcare system.

V06 – “... It was very challenging. And it’s kind of embarrassing because I’m like, ‘What? Who are you talking to?’ And they’re like, ‘Miss!’ You know like other than the person who initially brought me to the back, or put me in a stretcher or something, [she] doesn’t know that I’m blind. It gets back to what’s helpful.”

Additionally, introductions and identification are important for situational awareness for these patients to ensure their safety and basic needs are being addressed.

V01 – “At one point some food was left for me, but I didn’t know that it had been left there… you can’t see a person’s uniform or see their little badge that identifies them as an employee or what their name is so… if it could just be part of the training and part of the culture to say ‘Hi, my name is Mary. I’m from food service. I’m leaving your tray over here to the right’ or something, that would be really helpful.”

V06 – “… ‘Are you here to harm me or help me or what?’ You know, everybody from the doctor down to the essential floor sweeper, I’d like to know who they are and what they are intending to do.”

Participants also expressed discomfort when staff did not explain a procedure or task, especially if there was intrusive physical contact without preparation. Others went on to discuss the importance of clear instructions and descriptions prior to and during imaging procedures.

V01 – “Like if someone is going to give me a shot for instance. I can’t see it coming. So, I like for the doctor to say, ‘I’m going to give you an injection; this is what it’s for. It’s going to be in your left arm. I’m going to put some alcohol on you now.’ Otherwise, it just sort of happens out of the blue without warning because I’m not seeing the doctor doing the prep work in advance… before you do anything, just tell me what it is that you’re going to do, and that’s helpful… I think that just goes to the communications piece, knowing that a patient isn’t able to see any lights or read any signs; it really has to be verbal direction from staff members.”

Furthermore, participants emphasized the importance of respecting the patient’s autonomy and asking whether a patient wants assistance before offering it or touching them.

V04 – “Very rarely do people know to say, ‘I’ve noticed that you seem to be vision impaired,’ or ‘I’ve noticed that you’re using a cane; would you like a human guide?’ You know, they either take my arm or start guiding me by pushing my shoulder along or something like that.”

Participants repeatedly expressed the need for increased staff and volunteer training around sighted-guide (or human-guide) technique. The basis of the sighted-guide technique is to enable a person who is blind or has low vision to move through an environment safely with the assistance of a guide.^16^

V05 – “I would suggest that everyone, all the staff of the ED be trained [in sighted guide]… … And what [sighted guide] means is I would hold their elbow and then they would guide me and if there’s a step they’d say ‘step’ or ‘there’s a doorway over here.’ And not everyone is trained in that, but certainly a medical professional should be.”

For those with mobility challenges, patients face an additional barrier of navigating hallways with multiple obstructions, such as stretchers and hospital equipment that are designed for able-bodied personnel.

M14 – “When I’m having to walk with people they forget and they just keep walking and I might not be with them because I’m stuck. Like, transport often is unaware of the obstructions I’m dealing with.”

#### The need for electronic delivery of after-visit summaries for individuals with cognitive and visual disabilities

Participants expressed concern about the accessibility of documents they would receive in the ED, particularly related to discharge instructions or summaries.

V01 – “I think that the more forethought that a hospital can put into not only information, any information that a doctor would be distributing to a patient in the ER as a handout to take home, but also any kind of follow-up communication, it needs to be done in an accessible format.”

V05 – “My suggestion would be along with the normal whatever [after-visit summary] is given… if the instructions can be emailed… if the instructions were sent to me by email I could read them, no problem.”

Several participants shared the idea that larger print forms would be helpful for some patients with visual impairments.

V04 – [referring to discharge papers] “.. But in terms of what you go home with, it’s always pulling teeth. ‘Can you put this in large print for me?’ … And then it’s always 10 minutes of guiding them. ‘OK, you extract it and then you put it into a Word document and then you increase it to 32- point font… Stop looking at me like I’m a monster.”

#### The importance of mindful listening and patience by healthcare staff

Participants felt that patience was paramount when caring for PWD and appreciated more humanism in medicine. Participants emphasized human connection and keen listening.

M13 – “Sometimes I wish people would stop and take a breath and slow down and listen to the person more. Sometimes they’re so stressed and in a hurry. I don’t know. It’s very important to me to establish a human connection and sometimes people only have, you know, ‘Get these people in and out. Move fast, move fast.’ But you’re not servicing cattle; these are humans.”

Others spoke about how their disability impacts communication, or their ability to comply with medical directions, during an encounter.

M07 – “I know everybody is busy, but patience. Because I still lose my words. So sometimes you can’t get everything out, and before you can actually answer sometimes, they’re asking you another question… maybe they think you didn’t understand. I understood what you said, I just can’t get the words out!”

M09 – “One time where I had to get in a weird position, I did get in that position, but I was limited in how fast I could get into that position. And [staff] got a little irritated that it was taking me a little longer than some of you [able-bodied people].”

Others participants requested recognition of their autonomy and lived experience as a person with a disability.

M14 – “But it’s like we need… to be listened to because we are the ones who know our equipment. We know our bodies, we know our needs. We know our overlapping medical issues. We might be there for one problem, but you’re going to end up causing a different problem if you don’t listen to me and you don’t give me my regular meds that I need at this time. So I think from that standpoint, listening to those that are disabled, especially those with complex needs, we know ourselves the best. And that’s often under-recognized in medicine. Everybody wants to talk about us without us.”

#### The role of increased hospital support services including greeters and volunteers

Most participants recommended more volunteer services, specifically for navigation to and from the ED.

V05 – “ I think having somebody in the ED, if I didn’t have the family member there, if I had taken a Lyft [ride-share app], then the important thing would be for someone in the ED to see that you have some disability or can’t see… If I was alone, I would hope that somebody, some member of the ED staff, could help me kind of navigate the physical ED in order to get to the point where I could call the Lyft and kind of get me to the right place.”

Others noted volunteers would be helpful in meeting their basic needs such as going to the bathroom or getting comfort care items like a drink of water or warm blanket.

M07 – “So, I think that in situations like that, that’s an issue of dignity… I’m not just going to the bathroom to look in the mirror or something, I need to use the restroom, you know?... I’ve had it happen twice. Even though I was in bed the first time, I still couldn’t get anybody to take me to the restroom. So, it’s an issue of dignity?”

#### Comprehensive training with both prehospital and hospital staff around assistive devices and services

Participants shared that healthcare workers need to have increased training specifically around the proper use of assistive devices and services, such as wheelchairs, canes, and service animals.

M10 – “They told me to leave my cane folded up in the bag, like ‘don’t use that in [the ED]’… So they didn’t want me to use my cane or any of my devices, they didn’t want me to bring the rollator to the hospital, they didn’t want me to open the cane there, and they weren’t offering me like any other supplementary device or help, if I requested help, to get up!”

M14 – “It’s just always a technicality about everything. Automatically bring the stretcher. There’s no way to just know on a chart that goes to transport automatically to let somebody know that they’re a wheelchair user, and there’s a wheelchair to be used in some capacity … Or they would have to find a staff member willing to drive it from one building to the other. Which was always a nerve-wracking thing, in that I’ve got valuables on the chair, I don’t want to lose my chair.”

## DISCUSSION

This study highlights the experience of individuals living with disabilities to understand the barriers they face in the ED. We identified five key patient-centered areas for change that are actionable and feasible for any ED to implement. Prior research on healthcare access for individuals living with disabilities used a framework centered on seven core dimensions of accessibility.^17^ Our qualitative study revealed the dimensions of accommodation, acceptability, and awareness to be most applicable to understanding accessibility in the ED.

Accommodation remains the central tenet to many of the barriers and challenges facing patients living with disability when they visit the ED. Areas of improvement include sighted-guide training for all staff, electronic delivery of AVS, changes to patient transport policies to accommodate those with assistive devices and wheelchairs, and verbal descriptions of procedures and consent when working with visually impaired patients. Some participants reflected that when they requested accommodations from the healthcare staff, they felt ostracized or insulted. This finding is not unique to the ED, as prior research has found that even when accessible medical equipment is available, healthcare personnel are still hesitant to use it.^18^ Thus, it is important that any equipment or technology provided to improve accessibility be paired with healthcare worker training that enables personnel to feel comfortable using the equipment. Furthermore, prior studies have found that PWD desire improved accommodations for communication, navigating unfamiliar environments, and for completion of paperwork,^19^ all of which were concepts identified by participants in this study.

Acceptability and awareness also emerged as critical dimensions of healthcare accessibility for PWD, and analysis of these dimensions yielded results that we found to be unique to the ED. Suggestions for improving awareness and acceptability included the following: more consistent staff introductions when entering an exam room; visual reminders and signage to indicate a patient has a visual impairment; and assistance with entry, exit, and general navigation of the ED. It is our belief that improving global awareness of the needs of PWD is a unique challenge to the ED, where patients are being seen by unfamiliar clinicians and staff in an urgent context. Results of studies investigating the experiences of PWD in other fields, such as obstetrics and gynecology or primary care, have not highlighted the importance of staff introductions or signage to indicate disability.^5^, ^20^

It is likely that the pace of the ED, including rapid turnover of both patients and staff, influences the need for an improved communication infrastructure in this setting. Outside the hospital, interventions consisting of disability awareness training to improve disability awareness among members of the community have resulted in more positive emotional and cognitive attitudes toward individuals with disabilities.^21^ It is reasonable to believe that similar interventions conducted with hospital staff could help improve the emotional and cognitive awareness of PWD in ways that would engender a more caring and accepting environment.

The role of the ED as the catchment area that is open 24/7 has allowed it to remain accessible under other framework dimensions, including availability, geography, affordability, and timeliness. Additionally, healthcare facilities under the American Disability Act Standards for Accessible Design have created physical accommodations to ensure facilities are accessible to patients. However, this study highlights the need for more investment in staff training and expectations to ensure personnel are continuing to create an inclusive, accommodating environment for PWD.

## LIMITATIONS

This study had several limitations including its lack of generalizability, as patients were recruited locally. Additionally, the interviews were conducted remotely due to the COVID-19 pandemic, which limited our ability to access PWD, especially with the additional requirement of access to video call technology. We believe this also contributed to the small sample size and to challenges recruiting participants who were deaf or living with autism or intellectual disability. Our study was limited in scope as all of our participants had a physical disability, cognitive disability, or had blindness/visual impairment with limited engagement from other communities with disability. Future study should pursue understanding the perspectives of individuals from patients with deafness/hard of hearing and autism to understand the unique barriers to care for their populations.

## CONCLUSION

We investigated the experiences of individuals living with physical, cognitive, and visual impairments to better understand the barriers they face when receiving care in the ED. Common themes from interviews emerged, touching on many aspects of care that present challenges for patients living with disabilities. Improvements made to aspects of the ED relevant to these themes may lead to improved patient comfort and satisfaction, improved communication between ED staff and patients, and improved outcomes for patients living with disabilities.

## Figures and Tables

**Table 1:** Participant demographics

Characteristic	Subjects n (%)
Age (years)	
40–49	2 (16)
50–59	2 (16)
60–69	5 (41)
70–79	3 (25)
Gender	
Male	2 (16)
Female	10 (83)
Race	
White	8 (66)
Black	4 (33)
Asian	0
Other	0
Type of Disability	
Physical disability	6 (50)
Visual impairment	6 (50)
Cognitive impairment	2 (16)
Total	12 (100)
